# Penis Allotransplantation in Beagle Dog

**DOI:** 10.1155/2016/1489204

**Published:** 2016-02-08

**Authors:** Yongbin Zhao, Weilie Hu, Lichao Zhang, Fei Guo, Wei Wang, Bangqi Wang, Changzheng Zhang

**Affiliations:** Department of Urology, The General Hospital of Guangzhou Military Command, No. 111, Liuhua Road, Guangzhou, Guangdong 510010, China

## Abstract

This is an original research of penis allotransplantation. The paper presents an experiment allogenic penis transplantation model in Beagles, with a focus on recovery of blood supply and changes in tissue architecture. Twenty adult Beagles were allocated to 10 pairs for penile transplantation. After operation, the skin and glans were observed. If adverse symptoms occurred, the transplanted penis was resected and pathologically examined. Frequency of urination, urinary stream, and patency level were recorded 7 days after transplantation. Cystourethrography was performed on Day 10. The transplanted penises were resected on Day 14 for pathological examination. The research showed that transplanted penises survived after allotransplantation, and the dogs regained urination ability. Penis autotransplantation in Beagles is feasible. This preliminary study shows a potential for application of this new procedure for penis transplantation in humans.

## 1. Introduction

War injury, trauma, penis tumor surgery, and congenital diseases may be associated with partial or complete penile defect, which deprives men of the ability of urination while standing and sexual activity. This condition often causes mental and psychological trauma and induces a heavy emotional and psychological burden to the patients and their families. The main treatment methods for penis defects include phalloplasty with free flaps or pedicled flaps, penis lengthening, and penis reconstruction [1–3]. Ideal treatment should achieve excellent appearance and feeling, ability to urinate while standing, and successful sexual intercourse. The first case of penis replantation has been performed in 1971 [4], and has been followed by many others. However, replantation is not always possible and reconstruction is then necessary.

Resection of the superficial suspensory ligament and about one- to two-thirds of the deep suspensory ligament may be performed to increase the length of the penis, which could increase the length by about 3.2–5.0 cm (mean 4.1 cm) and 0.8–1.2 cm, respectively. However, there is a high risk of injuring the deep dorsal vein, dorsal artery, and nerves. Although this method could help patients with complete penile defect to regain the ability of standing urination, several issues remain including poor appearance of the glans penis, short penis length (<5.0 cm), and erectile dysfunction [5–8]. Recently, V-Y advancement flaps from the lower pubic area are being widely used in V-Y plasty to increase the length of penis [9, 10]. However, this approach also suffers from poor appearance of the glans penis, limited penile length, and erectile dysfunction.

Therefore, reproductive organ transplantation has been suggested as an important alternative treatment for penis defects. In a retrospective study, Lee et al. analyzed animal studies (including testicle transplantation, ovarian transplantation, and en bloc vagino-utero-ovarian transplantation) performed over the past 25 years and have proposed that allogeneic penis transplantation could be applicable in the near future if approved by ethics committees [11]. One patient in China successfully underwent penis allotransplantation in 2006, but the penis had to be resected because of psychological issues [12].

Penises from Brown-Norway rats were transplanted to adult Lewis rats (en bloc allogeneic transplantation), and results showed that immunosuppressors including FK506 could effectively prevent rejection in allogenic penis transplantation [13]. Transplantation of single organ including kidney, heart, liver, and lung has been widely applied, and the technologies involved are well known. Similar to limbs, the penis consists of skin, blood vessels, nerves, cavernous bodies, albuginea, and urethral mucosa, making penile transplantation a composite tissue transplantation [14], and the rejection reactions are usually more serious than in single organ transplantation [15, 16]. Results from animal studies have shown that penis autotransplantations had a high success rate; however, allogeneic penis transplantation still remains a challenge for clinicians [17, 18]. Penis defects do not endanger the life of the patients but cause profound psychological problems that greatly affect the patients' quality of life. Therefore, penis allotransplantation could be performed in selected cases with the appropriate immunosuppressive regimen.

The structures of the penis of Beagle dogs are very similar to the human penis [19]. In the present study, allogeneic penile transplantations using microsurgery were performed in Beagles to investigate the graft survival and the restoration of urination function, with a focus on recovery of blood supply and changes in tissue architecture. Results could provide evidence for allogeneic penile transplantation in humans.

## 2. Materials and Methods

### 2.1. Animals

Twenty Beagles (weight: 15–17 kg, mean 16.8 kg) were provided by Zhaoqing Kangda Experimental Animal Co., Ltd. The dogs had free access to water and food. The diameter of the dogs' penises ranged from 2.2 to 3.0 cm (mean 2.6 cm). The 20 dogs were allocated to 10 pairs, each pair consisting of half-brothers in order to reduce immunological issues and to focus on blood supply and changes in tissue architecture. This study was approved by the ethics committee of our hospital and was performed according to animal protection rules.

### 2.2. Microsurgical Allogeneic Penis Transplantation

Lymphocyte toxicity test [20] had to be negative before surgery. Muscular injection of scopolamine (0.3 mg) and phenobarbital sodium (0.1 g) was performed 30 min before surgery to decrease salivary gland secretion to help in performing tracheal intubation and maintain the respiratory tract unblocked. Heparin sodium (12,500 U) was intravenously injected to reduce the risk of embolism after surgery. Alternate muscular injection of Su-Mian-Xin II (0.08 mL/kg) and ketamine (5–10 mg/kg) was performed to induce general anesthesia. Iodine and alcohol were used to disinfect the skin. A U-shaped incision was made in the pubic area after draping. The cavernous body of the penis was isolated from the root of the scrotum, while the dorsal vessels and nerves were kept intact. The penis was resected at about 3 cm from the penile head, and a rubber band was used to block the blood flow. The resected penis was preserved in tissue protection fluid at 4°C for 30 min to remove the residual blood in the resected penis.

The bilateral dorsal veins and arteries and accompanying nerves were carefully isolated and protected under the assistance of a surgical microscope (magnification: 10x). About 2 cm of the vessels and nerves were isolated. Then, a clamp was used to block the vessels, and the tunica intima was rinsed using heparin sodium solution to prevent thrombosis. The residual cavernous body was ligated with a rubber band, and the dorsal vessels and nerves were isolated for about 2 cm. A custom-made 12 F silica gel induct catheter was inserted through the external urethral orifice into the bladder. Absorbable suture (4-0) was used to suture the cavernous body of urethra and albuginea of cavernous body of penis to restore the continuity of the penis and urethral canal. Continuous suture with two fixed points was performed to anastomose the deep dorsal vein of the penis using 9-0 damage-free vascular suture. Suture of the dorsal artery of the penis and accompanying nerves was made using 11-0 damage-free vascular suture. The rubber band was then loosened to identify any blood leakage of the vascular anastomosis and to observe the arterial pulse and vein filling. Size-4 thread was used to suture the subcutaneous tissue and size-7 thread was used to suture the penile skin. The incisions were covered by sterile gauzes and bound with elastic bandages.

### 2.3. Treatments and Observation

Combined immune induction was performed using preoperative FK506 (2 mg) and MMF (250 mg). Methylprednisolone (500 mg) and pantoprazole (80 mg) were used intraoperatively. Intravenous dripping of dexamethasone (7.5 mg/kg) was performed for 3 days (once/day) after surgery and then switched to 0.2 mg/kg/d of muscular injection for immune induction therapy. Maintenance immunosuppressive therapy using FK506 (1 mg/kg/d, intragastric administration) and MMF (20 mg/kg/d, intravenous dripping) was also performed. Ampicillin and bicillin were administered to prevent infection. Nutritional therapy and supporting treatment were also performed.

After surgery, the skin and glans were carefully observed to identify any color change and tissue swelling. The catheter was removed on Day 7 after the operation. Frequency of urination, urinary stream, and patency level were recorded. Cystourethrography was performed on Day 10. The transplanted penis was resected on Day 14 for pathological examination. If the glans was white, if the skin was black, if there was obvious tissue swelling or necrosis, or if there was an irreversible acute rejection during the observation period, the transplanted penis was resected immediately and pathological examination was performed. After the period of observation, all dogs were returned to the animal center for use in studies needing large animals (such as orthopedics or cardiac surgery), in accordance with the terms of the ethical approval.

### 2.4. Morphology of the Penile Cavernous Body

Anatomical structures of the penis cavernous body of adult Beagles (including cavernous body of the penis, cavernous body of urethra, blood supply, and nerves) were carefully observed. The number of penis cavernous bodies, number of dorsal penis veins, diameters of the bilateral dorsal veins, number of dorsal penis arteries, diameters of the bilateral dorsal arteries, and number of dorsal penis nerves were recorded.

### 2.5. HE Staining

Tissue sections were immersed in hematoxylin for 2 min, rinsed with distilled water for 1 min, immersed in eosin for 5 s, and rinsed with distilled water for 1 min. Section dehydration was performed using a series of alcohol concentrations (30 s for each concentration). Sections were cleared by xylene and mounted using neutral balsam.

## 3. Results

### 3.1. Morphology of the Penile Cavernous Body

**Figure 1:**
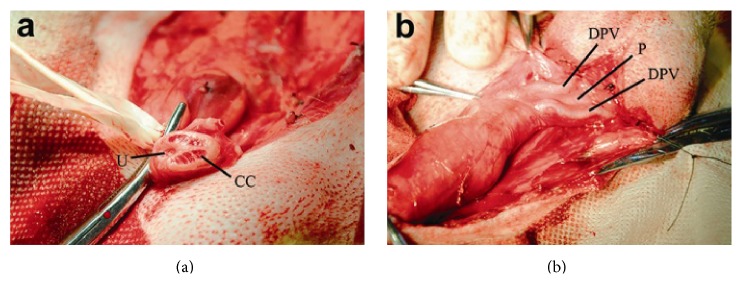
(a) Cross section of the cavernous bodies at the root of the penis of Beagles. (b) Dorsal penile vessels of Beagles. U: urethra; CC: corpus cavernosum; P: penis; DPV: dorsal penis vessels.

Skin, subcutaneous tissue, albuginea, and cavernous body could be clearly seen in the cross section of the penises. Cavernous bodies were separated by dense tissues, and the bilateral penile and urethral cavernous bodies were wrapped by albuginea. Blood sinuses of the cavernous bodies were regular and clear. Dorsal arteries, veins, and accompanying nerves could be found at dorsal penis. The mean length of the glans was 7.0 ± 1.5 cm, and the mean diameter of the penis was 2.0 ± 0.8 cm. Cartilage tissues were found in the penises. Penis cavernous bodies could be found from the root of the scrotum. Symmetric dorsal veins, arteries, and accompanying nerves were found at the dorsal penis. The mean diameter was 1.16 ± 0.11 mm for the left dorsal vein, 1.145 ± 0.11 mm for the right dorsal vein, and 0.46 ± 0.08 mm for both the left and right dorsal arteries (Figures 1(a) and 1(b), Table 1).

### 3.2. Survival and Morphology of the Transplanted Penises

For the 20 penis transplantations under the assistance of microsurgical techniques, the one-time success rate was 95% (38/40) for venous anastomosis and 87.5% (35/40) for arterial anastomosis. The mean vascular anastomosis time was 70.95 ± 8.95 min, mean operation time was 133.00 ± 10.31 min, and mean blood loss was 135.75 ± 41.40 mL (Table 2).

**Figure 2:**
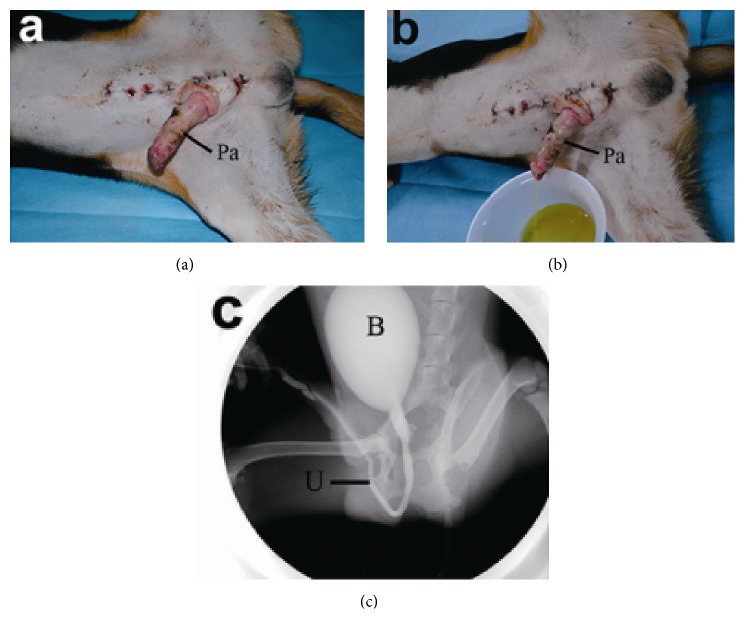
(a) The penis was with ruddy glans and mild pale penile skin, without swelling. (b) Normal urination of the transplanted penis. (c) Cystourethrography showing no urethrostenosis of the penis. Pa: penis allotransplant; B: bladder; U: urethra.

Among the 20 transplanted penises, the color of the glans and skin was pale for two penises on the first day after the operation, with tissue swelling. Glans necrosis, black skin, and mild tissue swelling were observed in three penises on Day 3. Black glans and skin and dry glans were observed in another three penises on Day 5, without swelling. The remaining 12 penises had a ruddy glans and mild pale penile skin, without swelling (Figure 2(a)). The incisions of these 12 penises recovered well, and the catheters were removed on Day 7. Normal urination and linear urinary stream were found (Figure 2(b)). No urethrostenosis was found by cystourethrography (Figure 2(c)).

### 3.3. Pathological Examination

**Figure 3:**
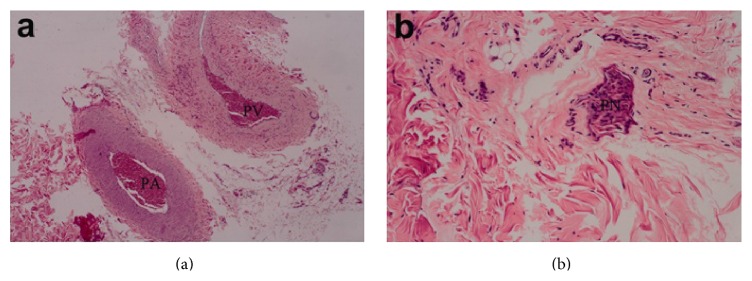
(a) Dorsal vein and artery of the penis in normal Beagles (magnification: 20x). (b) Penile nerve bundles in normal Beagles (magnification: 100x). PV: penis vein; PA: penis artery; PN: penis nerve.

Light microscopy examinations showed that the skin and subcutaneous tissues had normal structures, without interstitial edema. The vessel walls were clear, with numerous red blood cells inside. Nerve bundles were found surrounding the blood vessels (Figures 3(a) and 3(b)).

**Figure 4:**
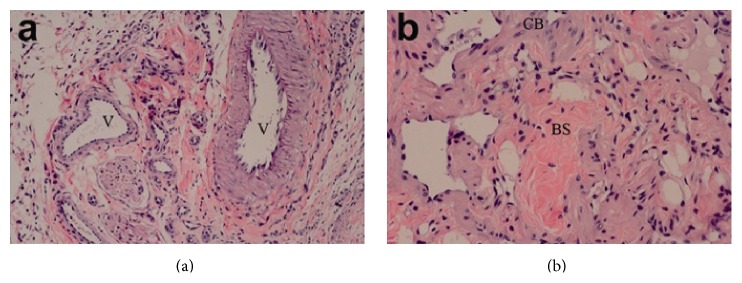
(a) No blood vessel embolism, and limited inflammatory cell infiltration (magnification: 100x). (b) Structure of the blood sinus in the cavernous body is normal, without degradation (magnification: 200x). V: vessels; CB: cavernous body; BS: blood sinus.

All successfully transplanted penises (*n* = 12) survived under immunosuppressive treatment. Normal structure of the skin and mild swelling of the subcutaneous tissues were found. No embolism was found in the blood vessels. The structure of blood sinus in the cavernous body was normal, with limited infiltration of inflammatory cells. No interstitial degeneration or necrosis was found (Figures 4(a) and 4(b)).

**Figure 5:**
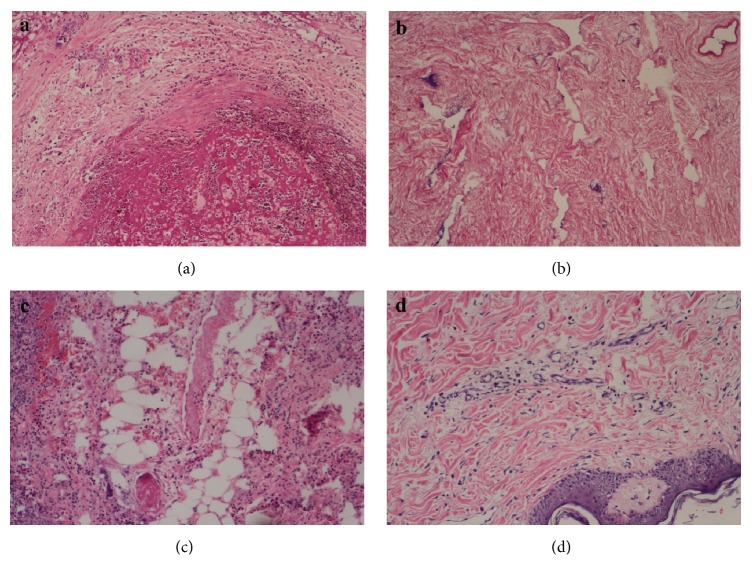
(a) Thrombosis in a vessel of a transplanted penis. Degradation can be seen in surrounding tissues (×100). (b) Structure disorder in the corpus cavernosum. The blood sinus disappeared, leading to tissue degradation (×100). (c) Inflammatory cell infiltration, vascular thrombosis, degradation, and edema in surrounding tissues (×100). (d) Inflammatory cell infiltration of the subcutaneous tissue and degradation (×100).

Eight penises were excised because of pale color or signs of failure.  Figure 5 presents the histopathological features of these penises, mainly including thrombosis, inflammatory cell infiltration, and tissue degradation.

## 4. Discussion

In two previous studies, penis transplantation was successfully performed in rat models [21, 22]. However, the structure of the rat's penis is different from human's. Therefore, large animal models with penises more similar to humans' are needed. The blood flow of penis cavernous bodies of adult Beagles is mainly from the cavernosal arteries, although they also receive minor blood supply from the dorsal vessels [23], which is more similar to human's than rat models. In addition, animals should not be too expensive. Therefore, Beagle could be an appropriate large animal model for the investigation of penis transplantation. Indeed, previous studies have shown that adult Beagles could be used as models for the transplantation of a number of organs including lung, kidney, and heart transplantation for preclinical testing of drugs or as proof of concept [24–26]. The findings of the present study demonstrated that penile allotransplantation with microsurgical techniques was possible in Beagles in the same way as in previous studies of transplantation of other organs.

In the present study, 12 of the 20 transplanted penises survived well, suggesting that immunosuppressive agents could effectively suppress immunological rejection in penis transplantation. Eight penises were cut because they were pale in color or showed signs of failure, but not all of them were necessarily failures. Supportive treatments might have rescued some of them, but we were aiming at observing the morphological changes. These changes mainly included thrombosis, inflammatory cell infiltration, and tissue degradation, all of which are associated with organ failure/rejection [27, 28]. In this study, the dorsal vessels were anastomosed, but it is likely that these failures might come from the inappropriate reconstitution of the blood supply from the external pudendal vessels. A recent study has shown that many penis transplants showed skin necrosis, probably for the same reason [23, 29]. Indeed, Tuffaha et al. [23] underline that the dorsal arteries are necessary for distal penis perfusion, but that the external pudendal artery is necessary for adequate skin perfusion. On the other hand, the studies by Tuffaha et al. [23, 29] were carried out using cadaveric penises, and it is unsure whether the anatomies are exactly the same between the human's and Beagle's penises. Additional studies are necessary to address this issue and to examine if skin necrosis could be avoided by external pudendal vessel anastomosis.

Previous studies on penis transplantation were performed in rats [21, 22], and no study was available in large animals. Therefore, we based our immunosuppressive regimen on these studies in rats and on our experience. Results showed that this regimen seemed adequate. However, future studies should be performed to address the most appropriate and optimal immunosuppressive regimen. Recent strategies in kidney transplantation have been shown to be efficient for composite tissue allograft and might be used for penis allotransplantation [30]. In a previous study performed by Koga et al., the survival rate of the transplanted penises was 100% after FK506 treatment, and rejection was minimal to moderate on Days 3 and 5 after transplantation and minimal or absent on Days 7, 10, 14, and 21 [13].

In 2006, our center reported a case of penile allotransplantation in a trauma patient. In that case, the deep dorsal vein, dorsal artery, and accompanying nerves were successfully anastomosed with microsurgical techniques, the cavernous body of the urethra and albuginea of the cavernous body was sutured, and a 16 F double-channel catheter was implanted. Maintenance immunosuppressive treatment with combined use of cyclosporine A, mycophenolate mofetil, and prednisone was performed after surgery. The blood supply of the transplanted penis was adequate, and the catheter was removed on day 10. No rejection reaction or infection was found, and the patient could urinate normally. However, despite technical success, the penis was resected on Day 14 due to the psychological repellence of the patient and his family [12]. This repellence could not be predicted, but psychological counseling might have prevented it. This will be an issue to address in eventual clinical trials. Nevertheless, it could be noted by its color that the skin of the penis was maybe suffering from an inadequate blood supply, which might be due to improper anastomosis of the external pudendal arteries [23, 29]. Again, as for the Beagles, additional studies are necessary to address this issue.

The advancement of several technologies including transplantation immunity, tissue typing and immunosuppressants, the high long-term survival rate of extremity allotransplantation in animals, and the high success rate of penis allotransplantation in the present study provides a solid ground for treating patients with penile defect using penis transplantation. Penis defects do not endanger the life of the patients but cause profound psychological problems that greatly affect the patients' quality of life. Therefore, we believe that if using a proper immunosuppressive regimen, penis transplantation could be ethical in selected cases. In a similar manner, disfigurement does not endanger life, but a recent bioethics analysis has shown that there are valid arguments for facial transplantation despite the risks associated with the procedure [31]. Similar arguments could be applied to penis transplantation. Erectile function was not assessed in the present study but will be in a future study.

## 5. Conclusion

The present study strongly suggests that the structure of the penis cavernous body is highly similar in Beagles compared with humans. Therefore, penis allotransplantation could be successfully performed with microsurgical techniques. Adult Beagles could be used as an experimental model for the investigation of penis allotransplantation.

## Figures and Tables

**Table 1:** Anatomic features of the penis from adult Beagles.

Parameters	Value
*n*	20
Mean number of cavernous bodies	3 ± 0
Mean number of dorsal penile veins	2 ± 0
Mean diameter of dorsal penile veins (mm)	
Left	1.16 ± 0.11
Right	1.145 ± 0.11
Mean number of dorsal penile arteries	2 ± 0
Mean diameter of dorsal penile arteries (mm)	
Left	0.46 ± 0.08
Right	0.46 ± 0.08
Mean number of dorsal penile nerves	
Left	1.35 ± 0.49
Right	1.30 ± 0.47

**Table 2:** Parameters of penis transplantations.

Parameters	Value
*n*	20
Number of dorsal veins	40
Number of one-time successes	38
Success rate	95%
Number of dorsal arteries	40
Number of one-time successes	35
Success rate	87.5%
Vascular anastomosis time (min)	70.95 ± 8.95
Operation time (min)	133.00 ± 10.31
Blood loss volume (mL)	135.75 ± 41.40
